# Experimental Strategies to Explore Drug Action and Resistance in Kinetoplastid Parasites †

**DOI:** 10.3390/microorganisms8060950

**Published:** 2020-06-24

**Authors:** Magali Van den Kerkhof, Yann G.-J. Sterckx, Philippe Leprohon, Louis Maes, Guy Caljon

**Affiliations:** 1 Laboratory of Microbiology, Parasitology and Hygiene (LMPH), University of Antwerp, 2610 Wilrijk, Belgium; Magali.Vandenkerkhof@uantwerpen.be (M.V.d.K.); Louis.Maes@uantwerpen.be (L.M.); 2 Laboratory of Medical Biochemistry (LMB), University of Antwerp, 2610 Wilrijk, Belgium; Yann.Sterckx@uantwerpen.be; 3 Centre de Recherche en Infectiologie du Centre de Recherche du Centre Hospitalier Universitaire de Québec, Université Laval, Québec, QC G1V 0A6, Canada; philippe.leprohon@crchudequebec.ulaval.ca

**Keywords:** kinetoplastid, resistance, target, drug, omics, mechanism, resistance

## Abstract

Kinetoplastids are the causative agents of leishmaniasis, human African trypanosomiasis, and American trypanosomiasis. They are responsible for high mortality and morbidity in (sub)tropical regions. Adequate treatment options are limited and have several drawbacks, such as toxicity, need for parenteral administration, and occurrence of treatment failure and drug resistance. Therefore, there is an urgency for the development of new drugs. Phenotypic screening already allowed the identification of promising new chemical entities with anti-kinetoplastid activity potential, but knowledge on their mode-of-action (MoA) is lacking due to the generally applied whole-cell based approach. However, identification of the drug target is essential to steer further drug discovery and development. Multiple complementary techniques have indeed been used for MoA elucidation. In this review, the different ‘omics’ approaches employed to define the MoA or mode-of-resistance of current reference drugs and some new anti-kinetoplastid compounds are discussed.

## 1. Kinetoplastid Diseases

Leishmaniasis, Chagas disease, and sleeping sickness are caused by kinetoplastid protozoan parasites and are responsible for high morbidity and mortality rates, especially in developing countries [1,2,3]. These diseases are characterized by severe clinical manifestations such as hepatosplenomegaly, cardiomyopathy, and neuropathology, all of which may lead to fatality if left untreated [4,5,6]. Current therapies are known to be less than adequate due to the suboptimal administration routes and long treatment duration, the occurrence of severe adverse effects, and the growing incidence of treatment failures [3,5,7]. It is evident that treatment options should be improved and that new drugs will be needed to sustain adequate disease control.

Target-based and phenotypic screening are two standard approaches adopted by the pharmaceutical industry to identify novel active chemical entities. So far, the former has not been very successful given the lack of fully validated targets and the limited knowledge on their molecular biology [3,4,8]. The few targets that have been suggested for *Trypanosoma brucei* and *T. cruzi* have only been partially characterized [8,9,10,11,12] and ornithine decarboxylase is currently the only fully validated target involved in the mode-of-action (MoA) of eflornithine [8,13]. Identification of novel drug ‘leads’ is generally still achieved by phenotypic cell-based screening [14,15,16,17] but with the disadvantage of lack of knowledge gained on the MoA. However, target elucidation remains pivotal for rational structure-based optimization of small molecules, which predicts adverse effects. Even though MoA studies are not essential for regulatory approval, they can strengthen successful drug development or redirect drug discovery [12,14,18,19,20]. Alternatively, knowledge on a drug’s mode-of-resistance (MoR) can also provide useful information for anti-kinetoplastid drug research [21].

**Figure 1:**
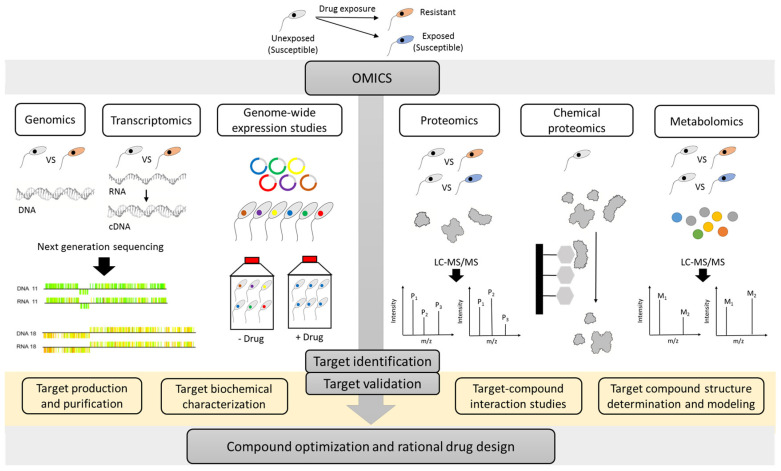
An overview of the multiple omics strategies that can be used to unravel the target of an anti-kinetoplastid compounds. Resistant parasites can be experimentally selected in the laboratory or obtained from the field. Comparative genomics, transcriptomics, proteomics/metabolomics approaches, genome-wide cosmid/RNAi libraries, and chemical proteomics can contribute complementary insights in the mode-of-action (MoA) or mode-of-resistance (MoR). Application of the various omics methodologies can enable the identification of drug targets, which may serve as starting point for additional hit finding, lead optimization and rational drug design. M: metabolite and P: protein. A graphical item in the figure about next generation sequencing was adopted from another publication [23].

As described in several reviews [18,19,22], multiple strategies can be used to identify the MoA of compounds. The present review specifically focuses on the different ‘omics’ approaches which in recent years have emerged as valuable techniques to evaluate the MoA and MoR of anti-kinetoplastid compounds (Figure 1).

## 2. Genomics

Forward genetics generally involve the identification of the gene(s) responsible for a resistant phenotype by performing a comparative study between a resistant line and its parent wild type [24]. For MoA/MoR studies of new chemical entities, an untargeted approach by next generation sequencing (NGS) is mostly used (Table 1) (reviewed for *Leishmania* by Leprohon et al. 2015 [25]). In general, whole genome sequencing (WGS) allows the detection of mutations such as single nucleotide polymorphisms (SNPs), as well as insertions and deletions (Indels), which can lead to resistance when occurring in drug targets or transporters [26,27,28,29,30,31,32]. Additionally, copy number variations (CNV) do occur in kinetoplastids and should therefore be incorporated in the analysis of altered drug tolerance [33,34,35,36,37,38,39]. CNVs arise as a result of the peculiar mechanism of genetic regulation in kinetoplastids where individual gene promotors are lacking and transcriptional regulation relies on RNA stability, resulting in the development of alternative tools to increase or decrease gene expression [40,41,42,43]. This could even lead to whole chromosome copy number variations (CCNV) which have been observed in laboratory strains of *Leishmania* and *T. cruzi* [43,44,45,46,47,48,49,50,51]. These structural alterations are particularly observed in in vitro expanded parasites, suggesting that this phenomenon might be a short-term adaptation mechanism to external stress and to an in vitro culture environment [35,46,52]. On the other hand, *T. brucei* does not display karyotype alterations probably due to a different DNA replication and recombination mechanism [43,53]. The use of DNA microarrays, also known as comparative genomic hybridization assays (CGH), has allowed for the successful identification of karyotypic alterations in drug-resistant parasites [23,35,45,54,55]. Resistant strains are generally obtained upon in vitro selection on axenic parasites by stepwise increase of drug pressure. In some cases, mutagenesis prior to selection may generate more resistant mutants [26,56,57,58,59,60]. Selection on axenic stages has the advantage of being simple and relatively fast but lacks biological relevance in *T. cruzi* and *Leishmania* as these are intracellular pathogens in the vertebrate host. Accepting that this may not be a primary issue in MoA studies, it should be kept in mind that confirmation in the intracellular stage will ultimately be needed [61,62]. The downside of experimental resistance selection is that a lot of spontaneous mutations occur because of the high continuous drug pressure or the prior mutagenesis, further complicating the identification of the actual gene(s) responsible for the loss in drug susceptibility [50,63]. The use of multiple parallel resistant strains could help in finding the needle in the haystack by identifying commonly mutated gene(s) [27,32,46,55,56,64,65].

## 3. Transcriptomics

Another technology that has frequently been used to elucidate the MoR in kinetoplastids is comparing the transcriptome of resistant and susceptible parasites (Table 2). Untargeted transcriptomics to compare treated versus untreated parasites was only performed once in *T. cruzi* [78]. However, the evaluation of mRNA transcript abundance in kinetoplastids has mostly been used in fundamental biology studies evaluating changes in expression between the various life cycle stages or to evaluate host-pathogen interactions [79,80,81,82,83,84,85,86]. The measurement of RNA levels can provide a broad range of information, but it should be kept in mind that gene expression in kinetoplastids relies on post-transcriptional regulation. Protein coding genes are transcribed as long polycistronic genes and are further processed into mature mRNAs by trans-splicing and polyadenylation [87,88]. Further post-transcriptional control relies on various mechanisms such as mRNA stability, translation, and degradation [40,89,90], often resulting in a weak correlation between mRNA and protein abundance. This discrepancy is considered one of the main disadvantages of transcriptomics in kinetoplastids [90]. As already mentioned, CNVs on chromosome or gene level are common events in kinetoplastids as one of their tactics to overcome the lack of transcriptional control. This modulation of RNA levels can often be ascribed to gene amplification or deletion events [33,35,91,92]. In some cases, elevated RNA levels could not be linked to CNVs and therefore probably relate to increase in stability [33,35,50,91,93,94]. Microarrays have been used to study the transcriptome but has a number of disadvantages, such as the high initial cost to construct specific chips for one of the kinetoplastids, as well as problems involving cross-hybridization and the inability to distinguish closely related genes. There is also concern about the lack in sensitivity and the possibility to miss low abundant genes [80,95,96,97]. More recently, high-throughput cDNA sequencing technology, also known as RNA-seq, has been developed [98]. This technique is fast, reliable, and allows the accurate quantification of transcript levels with high reproducibility and little technical variability [80,99]. In addition, RNA-seq provides a greater dynamic range compared to classical microarrays, thereby yielding more accurate information on the relative levels of transcription for genes that are expressed at very low or very high levels. Drawbacks are similar as those for WGS and include high cost, complex data analysis, and the need for adequate read depth [98,100,101]. To conclude, transcriptomics has increased the understanding of transcriptional control in kinetoplastids and can be an informative tool in resistance studies by identifying differentially expressed genes that could play a role in the decreased susceptibility.

## 4. Genome-Wide Expression Studies

The use of genome-wide overexpression or knockdown studies allows the identification of genes that may be involved in the MoA/MoR of drugs without the need of a time-consuming resistance selection process (Table 3). In general, genome wide knockdown studies will allow the identification of genes involved in drug uptake or activation, while overexpression studies identify the protein-binding target itself or genes that are involved in drug efflux or detoxification [108,109,110]. A cosmid-based gain-of-function screen has been developed in *Leishmania* and has recently been combined with NGS, also known as Cos-seq, allowing a full and unbiased view of the enriched genes [108]. Multidrug resistance associated protein A (MRPA), antimony (Sb) resistance marker 58 (ARM58), and heat shock proteins (HSPs) are a few examples of genes that have been identified when exposing cosmid libraries to Sb [108,111,112,113]. Additionally, this technique has confirmed N-myristoyltransferase as a drug target for pyrazolyl sulfonamide (PS) [114]. In some cases, cosmid libraries are transfected in resistant lines to identify the dominant negative mutations that underlie the resistant phenotype [26,115]. Yet another study combined the use of both susceptible and resistant strains to prepare and transfect the cosmid libraries [111]. All these studies have used the extracellular promastigote stage as it is known to be easier and faster. Only two studies opted to use intracellular amastigotes for the selection of cosmid bearing parasites [113,116], resulting in the identification of predominantly different genes compared to those identified in promastigotes [116]. These differences could be caused by a number of factors, not necessarily directly related to the drug, such as the host cell environment, parasite infectivity and intracellular multiplication [116]. For *T. brucei*, a tetracycline inducible overexpression library was developed and was able to confirm the drug target for difluoromethylornithine (DFMO), a N-myristoyltransferase inhibitor (N-MT), and even a novel benzoxaborole (BOX) [110,117]. Even though this strategy is promising, most of the genome wide expression screens in *T. brucei* are RNA interference (RNAi)-mediated knockdown studies. The use of these loss-of-function screens has been validated by using current anti-trypanosomal drugs all leading to the identification of known drug transporters or activators, such as amino acid transporter 6 (AAT6) for eflornithine (EFL), and nitro-reductase (NTR) for benznidazole (BZN) and nifurtimox (NFX) [109,118,119]. High-throughput detection of the phenotypes by NGS has been employed and is especially useful when more than a few targets are expected to play a role [120]. This technique is referred to as RNA interference target sequencing, also known as RIT-seq. Sadly, whole genome RNAi mediated knockdown studies are not available for *T. cruzi* and *Leishmania* spp. since they lack the genes encoding for the RNAi machinery, with the exception of the *L. viannia* subgenus [121]. For these species, the CRISPR/Cas9 technology could be an alternative tool to generate a loss-of-function library [122,123]. Some drawbacks of genome-wide expression studies are the inability to identify non-protein targets or drugs with complex multifactorial effects [110]. Knockdown of essential genes can also result in cell death reducing the chances of identifying such key genes in a RNAi screen [124]. At last, it should be taken into consideration that selection may favor parasites with a faster growth rate or increased infectivity [116,125]. To avoid false negative results, a good genome coverage is crucial and should be evaluated prior to selection [120].

## 5. Proteomics

In kinetoplastids, proteomics have been used to understand the differentiation processes in the various life cycle stages, to identify biomarkers, to support vaccine development, as well as to elucidate the MoA/MoR of drugs (Table 4) [85,138,139,140,141]. Several techniques can be used to compare the relative protein expression, but most employ labelling techniques such as 2D differential gel electrophoresis (2D-DIGE), stable isotope labeling of amino acids in cell culture (SILAC), or isobaric tags for relative and absolute quantitation (iTRAQ) [18,142]. Disadvantages of these labelling techniques are their high cost, the need of large amounts of protein fractions, the tedious sample preparation, and the high complexity of the acquired MS data [143,144]. An alternative is the use of label-free quantitative proteomics where relative protein abundance is determined by comparing peak intensities or number of tandem spectra between separately obtained LC-MS/MS runs [143,145,146]. 2D-DIGE is the mostly used technique in the study of MoR/MoA in kinetoplastids but is known to predominantly detect soluble and abundant proteins [139,146,147]. A way to evaluate a broader spectrum of the proteome is to additionally extract proteins from various specific cellular compartments or the use of differential extraction [138,139,148,149], such as the separate isolation of membrane-enriched and cytosolic proteins or the isolation of phosphoproteins, mitochondrial components, or the analysis of the secretome [68,150,151,152,153,154,155,156]. Most studies employing proteomics, especially in *Leishmania*, compared sensitive to resistant strains to elucidate the MoR of drugs. In contrast, most studies in *T. cruzi* and *T. brucei* compared treated and untreated samples to evaluate the MoA, resulting in a large number of possibly involved proteins that need further mining and follow-up studies to confirm their actual involvement [79,85]. The extracellular parasite stages are mostly used in these proteomics studies, which is not ideal as the proteome may differ depending on the life cycle stage [157,158,159,160]. Some of the technological hurdles include the identification of proteins that are still unannotated [138,161] and the identification of proteins that could be differentially expressed purely due to stress response and not due to the resistant phenotype or drug exposure [112,150,162,163].

## 6. Chemical Proteomics

Another way to analyze the proteome is to use affinity-based techniques where protein targets can be identified by the direct interaction with the compound/drug of interest [18,19,142,184,185,186]. This technology shows added value in the identification of the possible drug target(s) in kinetoplastids, especially for *T. brucei* (Table 5). Affinity techniques usually entail the need to chemically modify the small molecules to enable immobilization on a matrix, a process that can be lengthy and tedious and is not always fail-proof as immobilization of the drug may impede or alter target binding [18,142]. Non-specifically bound proteins are often detected but this can be avoided by the inclusion of appropriate experimental controls, e.g., using an inactive analogue bound to the matrix [30,142,187,188,189] or the use of free compounds in a competition experiment [30,73,187,190,191,192]. In some cases, SILAC has been employed to analyze these differential eluates to increase sensitivity and specificity, and to reduce technical variation [30,73,193]. An alternative to the standard affinity chromatography for the elucidation of drug targets is the addition of chemo- or photoreactive groups on small molecules to enable covalent binding to the target protein [191,192,194,195,196]. An added advantage of this technique is the possibility to incubate the probes in live cells instead of protein lysates [192,195,197]. When comparing differential proteomics (discussed above) with affinity-based techniques, the latter provides a more concise list of putative drug targets showing direct interaction with the small molecule. However, compounds with a low affinity to their target may be missed and the need of chemical modification is a hurdle and cannot always be applied [18]. A potential alternative is the use of label-free proteomics, such as drug affinity response target stability (DARTS), thermal protein profiling (TPP), or stability of proteins from rate of oxidation (SPROX), which are based on the idea that small molecules stabilize the target protein and therefore protect from respectively proteolysis, heat-denaturation or oxidation [185,198,199,200,201,202]. The use of TPP has recently confirmed N-myristoyltransferase as a drug target in *Leishmania* [114].

## 7. Metabolomics

Metabolomics enables the identification and quantification of many metabolites within a biological system as downstream product of gene, protein, and environmental interactions. As such, the metabolome can reflect the organism’s or cell’s physiological state and is closest to the phenotype [92,207]. Not only does this technique allow for the discovery or fine tuning of molecular mechanisms, it also allows for the identification of pathways involved in a drug’s MoA since drugs that inhibit enzymes cause alterations in the level of their substrates and products [18,62,208]. The experimental set-up is quite straightforward, where cells are grown in culture with and without drug and cell extracts are analyzed using mass spectrometry or nuclear magnetic resonance [62,161,209,210]. The drug exposure (duration and intensity) should be well chosen to allow the distinction between drug-specific effects and non-specific metabolic changes due to stress or secondary responses. Separation of the cellular extracts can be performed by liquid/gas chromatography or capillary electrophoresis, which are mostly complementary, to reduce complexity of the sample [62,161,209,211,212,213,214]. Metabolomics can provide an unbiased method to discover MoA pathways by comparing treated and untreated cell lines (Table 6) (reviewed by Creek et al. and Vincent et al. [62,215]). Alternatively, the comparison between susceptible and resistant strains can allow investigation of the MoR [66,70,72,74,211,216,217,218,219,220,221]. The primary challenge of the technique lays in the data deconvolution and in the identification of metabolites, as the kinetoplastid metabolomes have not yet fully been elucidated [215]. Even so, new pathways can be discovered, and compounds can still be classified based on their metabolic fingerprint [222,223,224].

## 8. Conclusions

Phenotypic screening has allowed the identification of new or repurposed chemical entities that display promising anti-kinetoplastid activity [14,15,16,17]. This cell-based strategy, however, does not give adequate insights into the MoA, which is useful for further compound optimization and overall success in drug development [14,18,19]. ‘Omics’ approaches offer tools to identify drug targets in an untargeted manner in an either direct (affinity) or indirect approach, resulting in the generation of a (long) list of potential target candidates. Although this may seem to be a disadvantage, combining results of complementary techniques permits fine-tuning of acquired data [18,30,56,114,232]. Target validation by either chemical or genetic means can further confirm a potential role in the MoA [19,120,233]. Importantly, it should be noted that such an undertaking often represents a first step in drug target validation and that additional experiments (such as enzymatic, biophysical and structural studies of the target-compound complex) plays a significant role in paving the road toward future optimization of the molecule by rational drug design. One major hurdle using ‘omics’ techniques is the functional annotation of genes, proteins and metabolites, since they are not always available for kinetoplastids [138,161,215]. Elucidation of the MoA is mostly based on the comparison between treated and untreated parasites or between resistant and susceptible strains. The latter will predominantly identify genes/proteins that are related to the MoR without necessarily providing information about the MoA. On the other hand, when comparing treated to untreated parasites, it is important to distinguish stress-related factors to the real drug effects by using adequate controls or an adapted drug concentration and exposure duration. Lastly, the kinetoplastids have multiple life cycle stages whereby different results can be obtained depending on the used parasite stage or form [116,192,234]. This should be kept in mind and additional confirmation in the host-relevant stage may be needed. In conclusion, a wide variety of complementary ‘omics’ tools are available for studies inquiring into drug actions and resistance mechanisms. While not always straightforward, their combination may enable novel target identification and trigger rational drug design.

## Figures and Tables

**Table 1:** Comparative whole genome sequencing (WGS) studies on laboratory-induced resistant strains to unravel the MoA or MoR of anti-kinetoplastid drugs. Experiments with *Leishmania* spp. and *T. cruzi* were performed on promastigotes and epimastigotes, respectively.

Organism	Drug/Compound	Main Relevant Gene(s)/Effect	Reference
*L. infantum*	AMB	MIL transporter	[66]
	AP	S-adenosylmethionine synthetase	[46]
	MIL *	MIL transporter	[28]
	MIL/PMM ^CM^	MIL transporter/ Protein kinase CDPK1	[67]
	Sb	Multiple	[68]
	SNF	S-adenosylmethionine transporter	[56]
*L. donovani*	AMB	24-sterol methyltransferase	[69]
	MIL	Multiple	[69]
	MIL	Multiple	[61]
	MIL	MIL transporter	[70]
	NIO	Nitroreductase 2	[71]
	PMM	D-lactate dehydrogenase like protein Aminotransferase branched chain amino acids	[69]
	PMM	Multiple	[72]
	PZP	Cyclin-dependent kinase 12	[73]
	Sb	Multiple	[69]
*L. major*	MIL	Miltefosine transporter Pyridoxal kinase α-adaptin like protein	[27]
	Sb	Aquaglyceroporin 1	[55]
*L. braziliensis*	Sb	Multiple	[50]
*L. guyanensis*	Sb	Aquaglyceroporin 1	[65]
*L. mexicana*	AMB	Sterol 14α-demethylase	[74]
	AMB	Sterol C24-methyltransferase	[75]
*L. panamanensis*	Sb	Multiple	[50]
*T. brucei*	BOX	Multiple	[76]
	FX	Nitroreductase	[63]
	MLP/PTM	Multiple	[64]
	NFX	Nitroreductase	[63]
	OX	Multiple	[30]
*T. cruzi*	BZN	Mutagenic	[39]
	GNF	Cytochrome B	[32]
	GNF	Proteasome inhibition	[31]
*T. congolense*	IC ^x^	Multiple	[77]

^x^ Resistance selection was performed in vivo. * Resistance selection was performed in the intracellular amastigote stage. ^CM^ Chemical mutagenesis was used prior to resistance selection. AMB: Amphotericin B, AP: Allopurinol, BOX: Benzoxaborole, BZN: Benznidazole, IC: Isometamidium chloride, FX: Fexinidazole, GNF: GNF-series, MEL: Melarsoprol, MIL: Miltefosine, NFX: Nifurtimox, NH: Nitroheterocyclics, NIO: Nitro-imidazo-oxazine, OX: Oxaborole PMM: Paromomycin, PTM: Pentamidine, PZP: Pyrazolopyrimidine, Sb: Antimonial, SNF: Sinefungin.

**Table 2:** Use of untargeted transcriptomics to unravel the MoR of drugs with anti-kinetoplastid effect by comparing resistant and susceptible parasites. Experiments with *Leishmania* spp. and *T. cruzi* were performed on promastigotes and epimastigotes, respectively.

Organism	Drug/Compound	Technique	Main Altered Expression	Reference
*L. infantum*	MTX	Whole genome microarray	Dihydrofolate reductase-thymidylate synthase ABCA_2_	[35]
	Sb	Whole genome microarray	Multidrug resistance protein A	[33]
*L. donovani*	AMB	RNA-seq	24-sterol methyltranferase	[69]
	MIL	Whole genome microarray	Multiple	[102]
	MIL	RNA-seq	Multiple	[61]
	MIL	RNA-seq	Multiple	[69]
	NFV °	Whole genome microarray	ABC transporters	[23]
	PMM	RNA-seq	D-lactate dehydrogenase like protein Aminotransferase branched chain amino acids	[69]
	Sb ^#^	Whole genome microarray	Histones Mitogen-activated protein kinase 1	[103]
	Sb ^#^	Genome microarray	ABC transporters	[97]
	Sb	RNA-seq	Multiple	[69]
*L. major*	MTX	Whole genome microarray	Dihydrofolate reductase-thymidylate synthase ABCA_2_	[35]
*L. braziliensis*	Sb	RNA-seq	Multiple	[50]
*L. mexicana*	AMB	RNA-seq	Sterol C24-methyltransferase	[75]
*L. panamensis*	Sb	RNA-seq	Multiple	[50]
*L. amazonensis*	Sb	Whole genome microarray	Multidrug resistance protein A Thiol metabolism	[94]
*T. brucei*	MLP/PTM	RNA-seq	Multiple	[64]
	SR	RNA-seq	Variant surface glycoprotein	[104]
*T. cruzi*	BZN ^#^	RNA-seq	Adenine phosphoribosyltransferase	[105]
	BZN ^#^	Genome microarray	ABCG_1_ transporter	[106]
	BZN ^x #^	Genome microarray	Old yellow enzyme	[107]

^x^ Resistance selection in vitro and in vivo. ° Resistance selection on axenic amastigotes. ^#^ Use of clinical resistant isolates. AMB: Amphotericin B, BZN: Benznidazole, MIL: Miltefosine, MLP: Melarsoprol, MTX: Methotrexate, NFV: Nelfinavir, PMM: Paromomycin, PTM: Pentamidine, Sb: Antimonial, SR: Suramin.

**Table 3:** Genome-wide expression studies to unravel the MoR or MoA of anti-kinetoplastid drugs. Experiments with *Leishmania* spp. and *T. cruzi* were performed on promastigotes and epimastigotes, respectively.

Organism	Drug/Compound	Technique	Main Identified Genes	Reference
*L. infantum*	AMB/MIL/MTX/ PMM/PTM/Sb	Cos-seq	Multiple	[108]
	AMB/MIL/Sb *	Cos-seq	Multiple	[116]
	MIL	Cosmid library	Unannotated	[126]
	Sb ^§^	Cos-seq	Heat shock proteins Antimony resistance marker	[113]
	SNF	Cos-seq	Adomet synthetase	[56]
*L. donovani*	BZF	Cos-seq	None	[127]
	MIL	^R^Cosmid library	MIL Transporter	[26]
	PS	Cos-seq	N-Myristoyltransferase	[114]
*L. major*	MTX	Cosmid library	Dihydrofolate reductase-thymidylate synthase Pteridine reductase 1	[128]
	PTM	Cosmid library	P-glycoprotein	[129,130]
	TUB	Cosmid library	Toxic nucleoside resistance	[128]
*L. braziliensis ^#^* *L. peruviana*	Sb	^R/S^Cosmid library	Antimony resistance marker 58	[111]
*L. tarentolae*	MTX	Cosmid library	Biopterin and folic acid transporter	[131]
	Sb	Cosmid library	Heat shock proteins	[112]
	Sb	^R^Cosmid library	Aquaglyceroporin 1	[115]
*T. brucei*	AMB/MIL/PMM/Sb	RIT-seq	Multiple	[132]
	BOX	RIT-seq	Aldehyde dehydrogenase 3	[133]
	BOX	Overexpression library	Cleavage and polyadenylation specificity factor 3	[117]
	CHA	RNAi library	Mitochondrial carrier protein family 14	[134]
	DFMO	Overexpression library	Ornithine decarboxylase	[110]
	dTUB	RNAi library	P_2_ nucleoside transporter Adenosine kinase	[135]
	EFL/MLP	RNAi library	Amino acid transporter 6/Adenosine transporter 1	[119]
	EFL/MLP/NFX/PTM/SR	RIT-seq	Multiple	[109]
	EFL/NFX	RNAi library	Amino acid transporter 6/Nitroreductase	[118]
	MTX/RX	RIT-seq	Folate transporter	[136]
	N-MT	Overexpression library	N-myristoyltransferase	[110,117]
	PDE	RNAi library	cAMP response proteins	[60]
	TUB	RNAi library	Hexose transporter Hexokinase	[137]

^#^ Use of clinical resistant isolates * Intracellular amastigote selection. ^§^ Both promastigote and intracellular amastigote selection. ^R^ Transfection was performed in resistant strains. ^R/S^ Transfection was performed in both resistant and susceptible strains. AMB: Amphotericin B, ARS: Arsenite, BZF: Benzothiopene-flavonol, BOX: Benzoxaborole, CHA: Choline analog, DFMO: Difluoromethylornithine, dTUB: 3′-deoxytubercidin, EFL: Eflornithine, MIL: Miltefosine, MLP: Melarsoprol, MTX: Methotrexate, NFX: Nifurtimox, N-MT: N-myristoyltransferase inhibitor, PDE: Phosphodiesterase inhibitor, PMM: Paromomycin, PS: Pyrazolyl sulfonamide, PTM: Pentamidine, RX: Raltitrexed, Sb: Antimonial, SNF: Sinefungin, SR: Suramin, TUB: Tubercidin.

**Table 4:** Use of untargeted proteomics to unravel the MoA or MoR of anti-kinetoplastid drugs. Experiments with *Leishmania* spp. and *T. cruzi* were performed on promastigotes and epimastigotes, respectively. T/UT: comparison between treated and untreated parasites. R/S: comparison between resistant and susceptible parasites.

Organism	Drug/Compound	Comparison	Extracted Proteome	Sample Preparation Prior to LC-MS/MS	Main Identified Protein(s)/Affected Pathways	Reference
*L. infantum*	AMB	R/S	Cytosolic and membrane enriched proteins	SILAC, SDS-PAGE	Glycolysis Tricarboxylic acid cycle	[150]
	MIL ^#^	R/S	Whole parasite	2D-DIGE	Redox homeostasis Stress response	[164]
	MIL/Sb	R/S	Mitochondrial proteins	2D-DIGE	Pyruvate dehydrogenase Succinyl-CoA:3-ketoacid-coenzyme A transferase	[155]
	Sb	R/S	Cytosolic and membrane enriched proteins	SILAC, SDS-PAGE	Multidrug resistance protein A	[68]
	Sb °	R/S	Whole parasite	2D-DIGE	Argininosuccinate synthetase Kinetoplastid membrane protein 11	[165]
	Sb	R/S	Whole parasite	2D-DIGE	Antioxidant defense Stress response	[163]
*L. donovani*	AMB ^#^	R/S	Secretome	SDS-PAGE	Carbohydrate metabolism Stress response	[154]
	ARS	R/S	Whole parasite	2D-DIGE	Multiple	[166]
	α-DFMO	R/S	Whole parasite	iTRAQ, SCX chromatography	Free radical detoxification Polyamine and trypanothione metabolic proteins	[167]
	MIL ^#^	R/S	Whole parasite	2D-DIGE	Iron superoxide dismutase	[168]
	MIL	R/S	Whole parasite	2D-DIGE	Eukaryotic initiation factor 4A	[169]
	NIO	R/S	Whole parasite	SILAC, SDS-PAGE	Nitroreductase 2	[71]
	PMM	R/S	Whole parasite	SILAC, SDS-PAGE	Translation and vesicle-mediated trafficking	[170]
	Sb ^#^	R/S	Whole parasite	2D-DIGE	Heat shock protein 83 Small kinetoplastid calpain-related protein	[171]
	Sb ^#^	R/S	Whole parasite	iTRAQ, SCX chromatography	Glycolysis Stress response	[172]
	Sb ^#^	R/S	Cytosolic and membrane enriched proteins	2D-DIGE	Heat shock proteins ABC transporter	[151]
	STQ	R/S + T	Whole parasite and cell fractionation	2D-DIGE	Multiple	[173]
*L. major*	MTX	R/S	Whole parasite	2D-DIGE	Pteridine reductase 1	[162]
	Sb ^#^	R/S	Whole parasite	2D-DIGE	Multiple	[174]
*L. braziliensis*	Sb	R/S	Whole parasite	2D-DIGE	Antioxidant defense Stress response	[163]
	Sb	R/S T/UT	Phospho-proteins	2D-DIGE	Stress response Antioxidant defense	[153]
*L. panamensis*	Sb	R/S	Whole parasite and membrane enriched proteins	2D-DIGE	Stress response Metabolic and transport functions	[175]
*L. tropica*	Sb ^#^	R/S	Whole parasite	2D-DIGE	Activated protein kinase c receptor Prostaglandin f2-alpha synthase	[176]
	Sb ^#^	R/S	Mitochondrial proteins	SDS-PAGE	Multiple	[156]
*T. brucei*	CYM	R/S	Whole parasite	2D-DIGE	Putative nascent polypeptide associated complex subunit	[177]
	LPB	T/UT	Phospho-proteins	SILAC, IMAC	Vesicle transport Gene expression	[152]
	PP	T/UT	Whole parasite	IMAC	Flagellar pocket protein BILBO-1 Tb14-3-3-associated protein kinase	[178]
	SR	T/UT	Whole parasite	SILAC, SDS-PAGE	Multiple	[179]
*T. cruzi*	BZN ^§^	R/S	Whole parasite	2D-DIGE	Transcription and protein destination	[180]
	NIZ	T/UT	Whole parasite	2D-DIGE	Activated protein kinase C receptor Asparagine synthetase	[181]
	NIZ	T/UT	Whole parasite	2D-DIGE	Redox metabolism Energy production	[182]
	PIP	T/UT	Whole parasite	2D-DIGE	Tryparedoxin peroxidase Methionine sulfoxide reductase	[183]
	VDM	T/UT	Whole parasite	SDS-PAGE	Multiple	[78]

° Experiment used axenic amastigotes. ^#^ Clinical resistant samples were used instead of laboratory induced resistance. ^§^ Both in vitro and in vivo selected resistant strains were used. IMAC: FeCl3-charged metal affinity chromatography. AMB: Amphotericin B, ARS: Arsenite, BZN: Benznidazole, CYM: Cymelarsan, α-DFMO: DL-α-Difluoromethylornithine, LPB: Lapatinib, MIL: Miltefosine, MTX: Methotrexate, NIO: Nitro-imidazo-oxazine, NIZ: Naphtoimidazole, PIP: Piplartine, PMM: Paromomycin, PP: Pyrrolopyrimidine. Sb: Antimonial, SR: Suramin, STQ: Sitamiquine, VDM: Vanadium analogue.

**Table 5:** Use of chemical proteomics to unravel the MoA of anti-kinetoplastid drugs. Experiments with *Leishmania* spp. and *T. cruzi* were performed on promastigotes and epimastigotes, respectively.

Organism	Drug/Compound	Technique	Main Identified Target(s)	Reference
*L. donovani*	PMM	Affinity chromatography	Paraflagellar rod proteins Prohibitin	[170]
	PZP	SILAC and Affinity chromatography	Cdc-2-related kinases Cyclins	[73]
*L. major*	dAP	Affinity chromatography	Mitogen-activated protein kinases Cdc2-related kinases	[190]
*L. mexicana*	PVB	Affinity chromatography	Cyclin-dependent kinase Casein kinase 1	[189]
*T. brucei*	ARM	Photo-affinity labelling	X	[191,203]
	AZN	Activity-based probes	Cathepsin B-like protease	[195]
	B-THP-T	Photo-affinity labelling	F_o_F_1_-ATP synthase	[197]
	CYM	Affinity chromatography	Glycerol-3-phosphate dehydrogenase	[204]
	dAP	Affinity chromatography	Mitogen-activated protein kinases Cdc2-related kinases	[190]
	MPH	Affinity chromatography	Adenosine kinase	[188]
	NQ	Affinity chromatography	Polypharmacology	[205]
	OX	SILAC and Affinity chromatography	Polypharmacology	[30]
	VS	Activity-based probes	Cysteine peptidase precursor Cathepsin B-like protease	[192]
*T. cruzi*	BZN	Affinity chromatography	Aldoketo reductase Protein transport protein Sec23A	[206]
	PVB	Affinity chromatography	Cyclin-dependent kinase Casein kinase 1	[189]

X: no identification step performed. ARM: Artemisinin, AZN: Azadipeptide nitrile, B-THP-T: Bis-tetrahydropyran 1,4-triazole, BZN: Benznidazole, CYM: Cymelarsan, dAP: 2,4-Diaminopyrimidine, MPH: 4-[5-(4-phenoxyphenyl)-2H-pyrazol-3-yl]morpholine, NQ: Naphtoquinone derivatives, OX: Oxaborole, PMM: Paromomycin, PVB: Purvalanol B, PZP: Pyrazolopyrimidine, VS: Vinyl sulfone.

**Table 6:** Application of untargeted metabolomics to unravel the MoA of anti-kinetoplastid drugs. All references in this table compared the metabolome between treated and untreated samples. Experiments performed with *Leishmania* were performed on promastigotes unless mentioned otherwise.

Organism	Drug/Compound	Main Affected Pathway(s)	Reference
*L. infantum*	AN	Pentose Phosphate pathway Preiss-handler salvage pathway	[225]
	MIL	Internal lipid metabolism	[217]
	Sb	Oxidative stress	[216]
	Sb	Redox metabolism Amino acid metabolism	[211]
	SRT	Polyamine and trypanothione biosynthesis Tricarboxylic acid cycle Phospholipid and sphingolipid metabolism	[226]
*L. donovani*	MIL °	Sphingolipids and sterol metabolism	[212]
	MIL	Polyamine metabolism from arginine to trypanothione (ROS)	[213]
*L. major*	FU/FdU	Deoxynucleotide metabolism	[227]
	MIL	Sphingolipids and sterol metabolism	[212]
*L. mexicana*	AN	Pentose Phosphate pathway Preiss-handler salvage pathway	[225]
	BS °	Deoxynucleotide metabolism Oxidative stress	[228]
	FU/FdU	Deoxynucleotide metabolism	[227]
*L. amazonensis*	LPV	Sterol metabolism	[229]
*T. brucei*	BOX	Methionine metabolism	[223]
	BOX	S-adenosyl methionine Methylation	[76]
	EFL	Polyamine pathway	[230]
	FdU	Thimidylate synthase	[221]
	FOA/FU	RNA modification	[221]
	SNF	S-adenosyl methionine 5′-methylthioadenosine	[223]
	NFX	Carbohydrate and nucleotide metabolism	[230]
*T. cruzi*	BZN	Thiol metabolism	[231]

° Use of axenic amastigotes. AN: 6-aminonicotinamide, BOX: Benzoxaborole, BS: Bisabolane sesquiterpene, BZN: Benznidazole, EFL: Eflornithine, FdU: 5-fluoro-2′-deoxyuridine, FOA: 5-fluoroorotic acid, FU: 5-fluoruracil, LPV: Lopinavir, MIL: Miltefosine, NFX: Nifurtimox, Sb: Antimonial, SNF: Sinefungin, SRT: Sertraline.

## References

[1] Barrett M.P., Burchmore R., Stich A., Lázzari J.O., Frasch A.C., Cazzulo J.J., Krishna S. (2003). The trypanosomiases. Lancet.

[2] Hotez P.J., Fenwick A., Savioli L., Molyneux D. (2009). Rescuing the bottom billion through control of neglected tropical diseases. Lancet.

[3] Rao S.P.S., Barrett M.P., Dranoff G., Faraday C.J., Gimpelewicz C.R., Hailu A., Jones C.L., Kelly J.M., Lazdins-Helds J.K., Mäser P. (2018). Drug Discovery for Kinetoplastid Diseases: Future Directions. ACS Infect. Dis..

[4] Zulfiqar B., Shelper T.B., Avery V.M. (2017). Leishmaniasis drug discovery: Recent progress and challenges in assay development. Drug Discov. Today.

[5] Perez-Molina J.A., Molina I. (2018). Chagas disease. Lancet.

[6] Büscher P., Cecchi G., Jamonneau V., Priotto G. (2017). Human African trypanosomiasis. Lancet.

[7] Baker C.H., Welburn S.C. (2018). The Long Wait for a New Drug for Human African Trypanosomiasis. Trends Parasitol..

[8] Gilbert I.H. (2013). Drug Discovery for Neglected Diseases: Molecular Target-Based and Phenotypic Approaches. J. Med. Chem..

[9] Renslo A.R., McKerrow J.H. (2006). Drug discovery and development for neglected parasitic diseases. Nat. Methods.

[10] Scarim C.B., Jornada D.H., Chelucci R.C., De Almeida L., Dos Santos J.L., Chung M.C., Chin C.M. (2018). Current advances in drug discovery for Chagas disease. Eur. J. Med. Chem..

[11] Osorio-Méndez J.F., Cevallos A.M. (2019). Discovery and Genetic Validation of Chemotherapeutic Targets for Chagas’ Disease. Front. Microbiol..

[12] De Rycker M., Baragaña B., Duce S.L., Gilbert I.H. (2018). Challenges and recent progress in drug discovery for tropical diseases. Nature.

[13] Grishin N.V., Osterman A.L., Brooks H.B., Phillips M.A., Goldsmith E.J. (1999). X-ray Structure of Ornithine Decarboxylase from *Trypanosoma brucei*: The Native Structure and the Structure in Complex with α-Difluoromethylornithine. Biochemistry.

[14] Sykes M.L., Avery V.M. (2013). Approaches to Protozoan Drug Discovery: Phenotypic Screening. J. Med. Chem..

[15] Alves F., Bilbe G., Blesson S., Goyal V., Monnerat S., Mowbray C., Ouattara G.M., Pécoul B., Rijal S., Rode J. (2018). Recent Development of Visceral Leishmaniasis Treatments: Successes, Pitfalls, and Perspectives. Clin. Microbiol. Rev..

[16] Freitas-Junior L.H., Chatelain E., Kim H.A., Siqueira-Neto J.L. (2012). Visceral leishmaniasis treatment: What do we have, what do we need and how to deliver it?. Int. J. Parasitol. Drugs Drug Resist..

[17] Berninger M., Schmidt I., Ponte-Sucre A., Holzgrabe U. (2017). Novel lead compounds in pre-clinical development against African sleeping sickness. MedChemComm.

[18] Tulloch L., Menzies S., Coron R.P., Roberts M.D., Florence G.J., Smith T.K. (2017). Direct and indirect approaches to identify drug modes of action. IUBMB Life.

[19] Terstappen G.C., Schlüpen C., Raggiaschi R., Gaviraghi G. (2007). Target deconvolution strategies in drug discovery. Nat. Rev. Drug Discov..

[20] Moffat J.G., Vincent F., Lee J.A., Eder J., Prunotto M. (2017). Opportunities and challenges in phenotypic drug discovery: An industry perspective. Nat. Rev. Drug Discov..

[21] Hefnawy A., Berg M., Dujardin J.-C., De Muylder G. (2017). Exploiting Knowledge on *Leishmania* Drug Resistance to Support the Quest for New Drugs. Trends Parasitol..

[22] Azad M.A., Wright G.D. (2012). Determining the mode of action of bioactive compounds. Bioorganic Med. Chem..

[23] Kumar P., Lodge R., Raymond F., Ritt J.F., Jalaguier P., Corbeil J., Tremblay M.J. (2013). Gene expression modulation and the molecular mechanisms involved in Nelfinavir resistance in *Leishmania donovani* axenic amastigotes. Mol. Microbiol..

[24] Berg M., Mannaert A., Vanaerschot M., Van der Auwera G., Dujardin J.-C. (2013). (Post-) Genomic approaches to tackle drug resistance in *Leishmania*. Parasitology.

[25] Leprohon P., Fernandez-Prada C., Gazanion E., Monte-Neto R., Ouellette M. (2014). Drug resistance analysis by next generation sequencing in *Leishmania*. Int. J. Parasitol. Drugs Drug Resist..

[26] Perez-Victoria F.J., Gamarro F., Ouellette M., Castanys S. (2003). Functional cloning of the miltefosine transporter—A novel P-type phospholipid translocase from *Leishmania* involved in drug resistance. J. Boil. Chem..

[27] Coelho A.C., Boisvert S., Mukherjee A., Leprohon P., Corbeil J., Ouellette M. (2012). Multiple Mutations in Heterogeneous Miltefosine-Resistant *Leishmania major* Population as Determined by Whole Genome Sequencing. PLoS Negl. Trop. Dis..

[28] Mondelaers A., Sánchez-Cañete M.P., Hendrickx S., Eberhardt E., García-Hernández R., Lachaud L., Cotton J.A., Sanders M., Cuypers B., Imamura H. (2016). Genomic and Molecular Characterization of Miltefosine Resistance in *Leishmania infantum* Strains with Either Natural or Acquired Resistance through Experimental Selection of Intracellular Amastigotes. PLoS ONE.

[29] Ritt J.-F., Raymond F., Leprohon P., Légaré D., Corbeil J., Ouellette M. (2013). Gene Amplification and Point Mutations in Pyrimidine Metabolic Genes in 5-Fluorouracil Resistant *Leishmania infantum*. PLoS Negl. Trop. Dis..

[30] Jones D.C., Foth B.J., Urbaniak M.D., Patterson S., Ong H., Berriman M., Fairlamb A.H. (2015). Genomic and Proteomic Studies on the Mode of Action of Oxaboroles against the African Trypanosome. PLoS Negl. Trop. Dis..

[31] Khare S., Nagle A.S., Biggart A., Lai Y.H., Liang F., Davis L.C., Barnes S.W., Mathison C.J.N., Myburgh E., Gao M.-Y. (2016). Proteasome inhibition for treatment of leishmaniasis, Chagas disease and sleeping sickness. Nature.

[32] Khare S., Roach S.L., Barnes S.W., Hoepfner D., Walker J.R., Chatterjee A.K., Neitz R.J., Arkin M.R., McNamara C.W., Ballard J. (2015). Utilizing Chemical Genomics to Identify Cytochrome b as a Novel Drug Target for Chagas Disease. PLoS Pathog..

[33] Leprohon P., Légaré D., Raymond F., Madore É., Hardiman G., Corbeil J., Ouellette M. (2009). Gene expression modulation is associated with gene amplification, supernumerary chromosomes and chromosome loss in antimony-resistant *Leishmania infantum*. Nucleic Acids Res..

[34] Rogers M.B., Hilley J.D., Dickens N., Wilkes J., Bates P., Depledge D.P., Harris D., Her Y., Herzyk P., Imamura H. (2011). Chromosome and gene copy number variation allow major structural change between species and strains of *Leishmania*. Genome Res..

[35] Ubeda J.-M., Légaré D., Raymond F., Ouameur A.A., Boisvert S., Rigault P., Corbeil J., Tremblay M.J., Olivier M., Papadopoulou B. (2008). Modulation of gene expression in drug resistant Leishmania is associated with gene amplification, gene deletion and chromosome aneuploidy. Genome Boil..

[36] Segovia M. (1994). Leishmania gene amplification: A mechanism of drug resistance. Ann. Trop. Med. Parasitol..

[37] Ouellette M., Borst P. (1991). Drug resistance and P-glycoprotein gene amplification in the protozoan parasite Leishmania. Res. Microbiol..

[38] Ranade R.M., Gillespie J.R., Shibata S., Verlinde C., Fan E., Hol W.G.J., Buckner F.S. (2013). Induced Resistance to Methionyl-tRNA Synthetase Inhibitors in *Trypanosoma brucei* Is Due to Overexpression of the Target. Antimicrob. Agents Chemother..

[39] Campos M.C., Phelan J., Francisco A.F., Taylor M.C., Lewis M.D., Pain A., Clark T.G., Kelly J.M. (2017). Genome-wide mutagenesis and multi-drug resistance in American trypanosomes induced by the front-line drug benznidazole. Sci. Rep..

[40] Clayton C., Shapira M. (2007). Post-transcriptional regulation of gene expression in trypanosomes and leishmanias. Mol. Biochem. Parasitol..

[41] Clayton C. (2002). Life without transcriptional control? From fly to man and back again. EMBO J..

[42] Haile S., Papadopoulou B. (2007). Developmental regulation of gene expression in trypanosomatid parasitic protozoa. Curr. Opin. Microbiol..

[43] Reis-Cunha J.L., Valdivia H.O., Bartholomeu D.C. (2018). Gene and Chromosomal Copy Number Variations as an Adaptive Mechanism Towards a Parasitic Lifestyle in Trypanosomatids. Curr. Genom..

[44] Reis-Cunha J.L., Rodrigues-Luiz G.F., Valdivia H.O., Baptista R.D.P., Mendes T.A.D.O., De Morais G.L., Guedes R., Macedo A.M., Bern C., Gilman R.H. (2015). Chromosomal copy number variation reveals differential levels of genomic plasticity in distinct *Trypanosoma cruzi* strains. BMC Genom..

[45] Minning T., Weatherly D.B., Flibotte S., Tarleton R.L. (2011). Widespread, focal copy number variations (CNV) and whole chromosome aneuploidies in *Trypanosoma cruzi* strains revealed by array comparative genomic hybridization. BMC Genom..

[46] Yasur-Landau D., Jaffe C.L., David L., Doron-Faigenboim A., Baneth G. (2018). Resistance of Leishmania infantum to allopurinol is associated with chromosome and gene copy number variations including decrease in the S-adenosylmethionine synthetase (METK) gene copy number. Int. J. Parasitol. Drugs Drug Resist..

[47] Obado S., Taylor M.C., Wilkinson S.R., Bromley E.V., Kelly J.M. (2005). Functional mapping of a trypanosome centromere by chromosome fragmentation identifies a 16-kb GC-rich transcriptional “strand-switch” domain as a major feature. Genome Res..

[48] Sterkers Y., Lachaud L., Bourgeois N., Crobu L., Bastien P., Pagès M. (2012). Novel insights into genome plasticity in Eukaryotes: Mosaic aneuploidy in *Leishmania*. Mol. Microbiol..

[49] Downing T., Imamura H., Decuypere S., Clark T.G., Coombs G.H., Cotton J.A., Hilley J.D., De Doncker S., Maes I., Mottram J.C. (2011). Whole genome sequencing of multiple *Leishmania donovani* clinical isolates provides insights into population structure and mechanisms of drug resistance. Genome Res..

[50] Patino L.H., Imamura H., Cruz-Saavedra L., Pavia P., Muskus C., Méndez C., Dujardin J.C., Ramírez J.D. (2019). Major changes in chromosomal somy, gene expression and gene dosage driven by SbIII in *Leishmania braziliensis* and *Leishmania panamensis*. Sci. Rep..

[51] Franssen S.U., Durrant C., Stark O., Moser B., Downing T., Imamura H., Dujardin J.-C., Sanders M.J., Mauricio I., Miles M.A. (2020). Global genome diversity of the *Leishmania donovani* complex. eLife.

[52] Mannaert A., Downing T., Imamura H., Dujardin J.-C. (2012). Adaptive mechanisms in pathogens: Universal aneuploidy in *Leishmania*. Trends Parasitol..

[53] Almeida L.V., Coqueiro-Dos-Santos A., Rodriguez-Luiz G.F., McCulloch R., Bartholomeu D.C., Reis-Cunha J.L. (2018). Chromosomal copy number variation analysis by next generation sequencing confirms ploidy stability in *Trypanosoma brucei* subspecies. Microb. Genom..

[54] Brazas M., Hancock R.E.W. (2005). Using microarray gene signatures to elucidate mechanisms of antibiotic action and resistance. Drug Discov. Today.

[55] Mukherjee A., Boisvert S., Monte-Neto R., Coelho A.C., Raymond F., Mukhopadhyay R., Corbeil J., Ouellette M. (2013). Telomeric gene deletion and intrachromosomal amplification in antimony-resistant *Leishmania*. Mol. Microbiol..

[56] Bhattacharya A., Sharma M., Packianathan C., Rosen B.P., Leprohon P., Ouellette M., Pakkinathan C. (2019). Genomewide Analysis of Mode of Action of the S-Adenosylmethionine Analogue Sinefungin in *Leishmania infantum*. mSystems.

[57] Coelho A.C., Trinconi C.T., Senra L., Yokoyama-Yasunaka J.K., Uliana S.R. (2015). *Leishmania* is not prone to develop resistance to tamoxifen. Int. J. Parasitol. Drugs Drug Resist..

[58] Iovannisci D.M., Kaur K., Young L., Ullman B. (1984). Genetic analysis of nucleoside transport in *Leishmania donovani*. Mol. Cell. Boil..

[59] Bhattacharya A., Bigot S., Padmanabhan P.K., Mukherjee A., Coelho A., Leprohon P., Papadopoulou B., Ouellette M. (2020). New insights in the mode of action of anti-leishmanial drugs by using chemical mutagenesis screens coupled to next-generation sequencing. Microb. Cell.

[60] Gould M.K., Bachmaier S., Ali J.A.M., Alsford S., Tagoe D.N., Munday J., Schnaufer A., Horn D., Boshart M., De Koning H.P. (2013). Cyclic AMP Effectors in African Trypanosomes Revealed by Genome-Scale RNA Interference Library Screening for Resistance to the Phosphodiesterase Inhibitor CpdA. Antimicrob. Agents Chemother..

[61] Vacchina P., Norris-Mullins B., Abengózar M.A., Viamontes C.G., Sarro J., Stephens M.T., Pfrender M.E., Rivas L., Morales M.A. (2016). Genomic Appraisal of the Multifactorial Basis for In Vitro Acquisition of Miltefosine Resistance in *Leishmania donovani*. Antimicrob. Agents Chemother..

[62] Vincent I., Barrett M.P. (2015). Metabolomic-Based Strategies for Anti-Parasite Drug Discovery. J. Biomol. Screen..

[63] Wyllie S., Foth B.J., Kelner A., Sokolova A.Y., Berriman M., Fairlamb A.H. (2015). Nitroheterocyclic drug resistance mechanisms in *Trypanosoma brucei*. J. Antimicrob. Chemother..

[64] Graf F., Ludin P., Arquint C., Schmidt R.S., Schaub N., Renggli C.K., Munday J., Krezdorn J., Baker N., Horn D. (2016). Comparative genomics of drug resistance in *Trypanosoma brucei* rhodesiense. Cell. Mol. Life Sci..

[65] Monte-Neto R., Laffitte M.-C., Leprohon P., Reis P., Frézard F., Ouellette M. (2015). Intrachromosomal Amplification, Locus Deletion and Point Mutation in the Aquaglyceroporin AQP1 Gene in Antimony Resistant *Leishmania (Viannia) guyanensis*. PLoS Negl. Trop. Dis..

[66] Fernandez-Prada C., Vincent I., Brotherton M.-C., Roberts M., Roy G., Rivas L., Leprohon P., Smith T.K., Ouellette M. (2016). Different Mutations in a P-type ATPase Transporter in *Leishmania* Parasites are Associated with Cross-resistance to Two Leading Drugs by Distinct Mechanisms. PLoS Negl. Trop. Dis..

[67] Bhattacharya A., Leprohon P., Bigot S., Padmanabhan P.K., Mukherjee A., Roy G., Gingras H., Mestdagh A., Papadopoulou B., Ouellette M. (2019). Coupling chemical mutagenesis to next generation sequencing for the identification of drug resistance mutations in *Leishmania*. Nat. Commun..

[68] Brotherton M.-C., Bourassa S., Leprohon P., Légaré D., Poirier G.G., Droit A., Ouellette M. (2013). Proteomic and Genomic Analyses of Antimony Resistant *Leishmania infantum* Mutant. PLoS ONE.

[69] Rastrojo A., García-Hernández R., Vargas P., Camacho E., Corvo L., Imamura H., Dujardin J.-C., Castanys S., Aguado B., Gamarro F. (2018). Genomic and transcriptomic alterations in *Leishmania donovani* lines experimentally resistant to antileishmanial drugs. Int. J. Parasitol. Drugs Drug Resist..

[70] Shaw C.D., Lonchamp J., Downing T., Imamura H., Freeman T.M., Cotton J.A., Sanders M., Blackburn G., Dujardin J.C., Rijal S. (2016). In vitro selection of miltefosine resistance in promastigotes of *Leishmania donovani* from Nepal: Genomic and metabolomic characterization. Mol. Microbiol..

[71] Wyllie S., Roberts A., Norval S., Patterson S., Foth B.J., Berriman M., Read K.D., Fairlamb A.H. (2016). Activation of Bicyclic Nitro-drugs by a Novel Nitroreductase (NTR2) in *Leishmania*. PLoS Pathog..

[72] Shaw C.D., Imamura H., Downing T., Blackburn G., Westrop G.D., Cotton J.A., Berriman M., Sanders M., Rijal S., Coombs G.H. (2019). Genomic and Metabolomic Polymorphism among Experimentally Selected Paromomycin-Resistant *Leishmania donovani* Strains. Antimicrob. Agents Chemother..

[73] Wyllie S., Thomas M., Patterson S., Crouch S., De Rycker M., Lowe R., Gresham S., Urbaniak M.D., Otto T.D., Stojanovski L. (2018). Cyclin-dependent kinase 12 is a drug target for visceral leishmaniasis. Nature.

[74] Mwenechanya R., Kovářová J., Dickens N., Mudaliar M., Herzyk P., Vincent I., Weidt S.K., Burgess K.E., Burchmore R., Pountain A.W. (2017). Sterol 14α-demethylase mutation leads to amphotericin B resistance in *Leishmania mexicana*. PLoS Negl. Trop. Dis..

[75] Pountain A.W., Weidt S.K., Regnault C., Bates P., Donachie A.M., Dickens N.J., Barrett M.P. (2019). Genomic instability at the locus of sterol C24-methyltransferase promotes amphotericin B resistance in *Leishmania* parasites. PLoS Negl. Trop. Dis..

[76] Begolo D., Vincent I., Giordani F., Pöhner I., Witty M.J., Rowan T.G., Bengaly Z., Gillingwater K., Freund Y., Wade R.C. (2018). The trypanocidal benzoxaborole AN7973 inhibits trypanosome mRNA processing. PLoS Pathog..

[77] Tihon E., Imamura H., Broeck F.V.D., Vermeiren L., Dujardin J.-C., Abbeele J.V.D. (2017). Genomic analysis of Isometamidium Chloride resistance in *Trypanosoma congolense*. Int. J. Parasitol. Drugs Drug Resist..

[78] Mosquillo M.F., Smircich P., Lima A., Gehrke S.A., Scalese G., Machado I., Gambino D., Garat B., Pérez-Díaz L. (2020). High Throughput Approaches to Unravel the Mechanism of Action of a New Vanadium-Based Compound against *Trypanosoma cruzi*. Bioinorg. Chem. Appl..

[79] Geiger A., Simo G., Grébaut P., Peltier J.-B., Cuny G., Holzmuller P. (2011). Transcriptomics and proteomics in human African trypanosomiasis: Current status and perspectives. J. Proteom..

[80] Patino L.H., Ramírez J.D. (2017). RNA-seq in kinetoplastids: A powerful tool for the understanding of the biology and host-pathogen interactions. Infect. Genet. Evol..

[81] Cruz A.K., Castro F.F. (2019). Genome and transcriptome analyses of *Leishmania* spp.: Opening Pandora’s box. Curr. Opin. Microbiol..

[82] Alonso A., Larraga V., Alcolea P.J.A. (2018). The contribution of DNA microarray technology to gene expression profiling in *Leishmania* spp.: A retrospective view. Acta Trop..

[83] Cantacessi C., Dantas-Torres F., Nolan M.J., Otranto D. (2015). The past, present, and future of *Leishmania* genomics and transcriptomics. Trends Parasitol..

[84] Oliveira A.E.R., Grazielle-Silva V., Ferreira L.R., Teixeira S.M.R. (2020). Close encounters between *Trypanosoma cruzi* and the host mammalian cell: Lessons from genome-wide expression studies. Genomics.

[85] Tiberti N., Sanchez J.-C. (2018). Sleeping Sickness in the ‘Omics Era. Proteom. Clin. Appl..

[86] Kumari S., Kumar A., Samant M., Singh N., Dube A. (2008). Discovery of novel vaccine candidates and drug targets against visceral leishmaniasis using proteomics and transcriptomics. Curr. Drug Targets.

[87] Clayton C. (2019). Regulation of gene expression in trypanosomatids: Living with polycistronic transcription. Open Boil..

[88] Martínez-Calvillo S., Vizuet-de-Rueda J.C., Florencio-Martínez L.E., Manning-Cela R.G., Figueroa-Angulo E.E. (2010). Gene Expression in Trypanosomatid Parasites. J. Biomed. Biotechnol..

[89] Campbell D.A., Thomas S., Sturm N.R. (2003). Transcription in kinetoplastid protozoa: Why be normal?. Microbes Infect..

[90] Krämer S. (2012). Developmental regulation of gene expression in the absence of transcriptional control: The case of kinetoplastids. Mol. Biochem. Parasitol..

[91] Guimond C., Trudel N., Brochu C., Marquis N., El Fadili A., Peytavi R., Briand G., Richard D., Messier N., Papadopoulou B. (2003). Modulation of gene expression in *Leishmania* drug resistant mutants as determined by targeted DNA microarrays. Nucleic Acids Res..

[92] Kumar B., Prakash A., Ruhela R.K., Medhi B. (2014). Potential of metabolomics in preclinical and clinical drug development. Pharmacol. Rep..

[93] Haimeur A., Guimond C., Pilote S., Mukhopadhyay R., Rosen B., Poulin R., Ouellette M. (1999). Elevated levels of polyamines and trypanothione resulting from overexpression of the ornithine decarboxylase gene in arsenite-resistant *Leishmania*. Mol. Microbiol..

[94] Monte-Neto R., Coelho A.C., Raymond F., Légaré D., Corbeil J., Melo M.N., Frézard F., Ouellette M. (2011). Gene Expression Profiling and Molecular Characterization of Antimony Resistance in *Leishmania amazonensis*. PLoS Negl. Trop. Dis..

[95] Haydock A., Terrao M., Sekar A., Ramasamy G., Baugh L., Myler P.J. (2014). RNA-Seq Approaches for Determining mRNA Abundance in *Leishmania*. Adv. Struct. Saf. Stud..

[96] Casneuf T., Van de Peer Y., Huber W. (2007). In situ analysis of cross-hybridisation on microarrays and the inference of expression correlation. BMC Bioinform..

[97] Singh N., Almeida R., Kothari H., Kumar P., Mandal G., Chatterjee M., Venkatachalam S., Govind M.K., Mandal S.K., Sundar S. (2007). Differential gene expression analysis in antimony-unresponsive Indian kala azar (visceral leishmaniasis) clinical isolates by DNA microarray. Parasitology.

[98] Wang Z., Gerstein M., Snyder M. (2009). RNA-Seq: A revolutionary tool for transcriptomics. Nat. Rev. Genet..

[99] Marioni J., Mason C.E., Mane S.M., Stephens M., Gilad Y. (2008). RNA-seq: An assessment of technical reproducibility and comparison with gene expression arrays. Genome Res..

[100] Hitzemann R., Bottomly D., Darakjian P., Walter N.A.R., Iancu O., Searles R., Wilmot B., McWeeney S.K. (2012). Genes, behavior and next-generation RNA sequencing. Genes Brain Behav..

[101] Malone J.H., Oliver B. (2011). Microarrays, deep sequencing and the true measure of the transcriptome. BMC Boil..

[102] Kulshrestha A., Sharma V., Singh R., Salotra P. (2014). Comparative transcript expression analysis of miltefosine-sensitive and miltefosine-resistant *Leishmania donovani*. Parasitol. Res..

[103] Singh R., Kumar D., Duncan R., Nakhasi H.L., Salotra P. (2010). Overexpression of histone H2A modulates drug susceptibility in *Leishmania* parasites. Int. J. Antimicrob. Agents.

[104] Wiedemar N., Graf F., Zwyer M., Ndomba E., Renggli C.K., Cal M., Schmidt R.S., Wenzler T., Mäser P. (2017). Beyond immune escape: A variant surface glycoprotein causes suramin resistance in *Trypanosoma brucei*. Mol. Microbiol..

[105] García-Huertas P., Mejía-Jaramillo A.M., González L., Chávez O.T. (2017). Transcriptome and Functional Genomics Reveal the Participation of Adenine Phosphoribosyltransferase in *Trypanosoma cruzi* Resistance to Benznidazole. J. Cell. Biochem..

[106] Zingales B., Araujo R.G.A., Moreno M., Franco J., Aguiar P., Nunes S.L., Silva M.N., Ienne S., Machado C.R., Brandão A. (2015). A novel ABCG-like transporter of *Trypanosoma cruzi* is involved in natural resistance to benznidazole. Mem. Instit. Oswaldo Cruz.

[107] Murta S.M., Krieger M.A., Montenegro L.R., Campos F.F., Probst C.M., Avila A.R., Muto N.H., De Oliveira R.C., Nunes L.R., Nirdé P. (2006). Deletion of copies of the gene encoding old yellow enzyme (TcOYE), a NAD(P)H flavin oxidoreductase, associates with in vitro-induced benznidazole resistance in *Trypanosoma cruzi*. Mol. Biochem. Parasitol..

[108] Gazanion E., Fernandez-Prada C., Papadopoulou B., Leprohon P., Ouellette M. (2016). Cos-Seq for high-throughput identification of drug target and resistance mechanisms in the protozoan parasite *Leishmania*. Proc. Natl. Acad. Sci. USA.

[109] Alsford S., Eckert S., Baker N., Glover L., Sánchez-Flores A., Leung K.F., Turner D.J., Field M.C., Berriman M., Horn D. (2012). High-throughput decoding of antitrypanosomal drug efficacy and resistance. Nature.

[110] Begolo D., Erben E., Clayton C. (2014). Drug Target Identification Using a Trypanosome Overexpression Library. Antimicrob. Agents Chemother..

[111] Nühs A., Schäfer C., Zander D., Trübe L., Nevado P.T., Schmidt S., Arevalo J., Adaui V., Maes L., Dujardin J.-C. (2013). A novel marker, ARM58, confers antimony resistance to *Leishmania* spp. Int. J. Parasitol. Drugs Drug Resist..

[112] Brochu C., Haimeur A., Ouellette M. (2004). The heat shock protein HSP70 and heat shock cognate protein HSC70 contribute to antimony tolerance in the protozoan parasite *Leishmania*. Cell Stress Chaperon.

[113] Nevado P.T., Bifeld E., Höhn K., Clos J. (2016). A Telomeric Cluster of Antimony Resistance Genes on Chromosome 34 of *Leishmania infantum*. Antimicrob. Agents Chemother..

[114] Corpas-Lopez V., Moniz S., Thomas M., Wall R.J., Torrie L.S., Zander-Dinse D., Tinti M., Brand S., Stojanovski L., Manthri S. (2018). Pharmacological Validation of N-Myristoyltransferase as a Drug Target in *Leishmania donovani*. ACS Infect. Dis..

[115] Marquis N., Gourbal B., Rosen B., Mukhopadhyay R., Ouellette M. (2005). Modulation in aquaglyceroporinAQP1gene transcript levels in drug-resistant *Leishmania*. Mol. Microbiol..

[116] Fernandez-Prada C., Sharma M., Plourde M., Bresson E., Roy G., Leprohon P., Ouellette M. (2018). High-throughput Cos-Seq screen with intracellular *Leishmania infantum* for the discovery of novel drug-resistance mechanisms. Int. J. Parasitol. Drugs Drug Resist..

[117] Wall R.J., Rico E., Lukac I., Zuccotto F., Elg S., Gilbert I.H., Freund Y., Alley M.R.K., Field M.C., Wyllie S. (2018). Clinical and veterinary trypanocidal benzoxaboroles target CPSF3. Proc. Natl. Acad. Sci. USA.

[118] Baker N., Alsford S., Horn D. (2011). Genome-wide RNAi screens in African trypanosomes identify the nifurtimox activator NTR and the eflornithine transporter AAT6. Mol. Biochem. Parasitol..

[119] Burkard G.S., Jutzi P., Roditi I. (2011). Genome-wide RNAi screens in bloodstream form trypanosomes identify drug transporters. Mol. Biochem. Parasitol..

[120] Glover L., Alsford S., Baker N., Turner D.J., Sánchez-Flores A., Hutchinson S., Hertz-Fowler C., Berriman M., Horn D. (2014). Genome-scale RNAi screens for high-throughput phenotyping in bloodstream-form African trypanosomes. Nat. Protoc..

[121] Lye L.-F., Owens K., Shi H., Murta S.M.F., Vieira A.C., Turco S.J., Tschudi C., Ullu E., Beverley S.M. (2010). Retention and Loss of RNA Interference Pathways in Trypanosomatid Protozoans. PLoS Pathog..

[122] Shalem O., Sanjana N.E., Zhang F. (2015). High-throughput functional genomics using CRISPR–Cas9. Nat. Rev. Genet..

[123] Lander N., Chiurillo M.A. (2019). State-of-the-art CRISPR/Cas9 Technology for Genome Editing in Trypanosomatids. J. Eukaryot. Microbiol..

[124] Fairlamb A.H. (2012). Infectious disease: Genomics decodes drug action. Nature.

[125] Clos J., Choudhury K. (2006). Functional cloning as a means to identify *Leishmania* genes involved in drug resistance. Mini Rev. Med. Chem..

[126] Choudhury K., Zander D., Kube M., Reinhardt R., Clos J. (2008). Identification of a *Leishmania infantum* gene mediating resistance to and SbIII. Int. J. Parasitol..

[127] Borsari C., Jiménez-Antón M.D., Eick J., Bifeld E., Torrado J.J., Olías-Molero A.I., Corral M.J., Santarem N., Baptista C., Severi L. (2019). Discovery of a benzothiophene-flavonol halting miltefosine and antimonial drug resistance in *Leishmania* parasites through the application of medicinal chemistry, screening and genomics. Eur. J. Med. Chem..

[128] Cotrim P., Garrity L.K., Beverley S.M. (1999). Isolation of Genes Mediating Resistance to Inhibitors of Nucleoside and Ergosterol Metabolism in Leishmaniaby Overexpression/Selection. J. Boil. Chem..

[129] Coelho A.C., Beverley S.M., Cotrim P. (2003). Functional genetic identification of PRP1, an ABC transporter superfamily member conferring pentamidine resistance in *Leishmania major*. Mol. Biochem. Parasitol..

[130] Coelho A.C., Tosi L.R.O., Cotrim P. (2004). Mapping of a *Leishmania major* gene/locus that confers pentamidine resistance by deletion and insertion of transposable element. Rev. Instit. Med. Trop. São Paulo.

[131] Kündig C., Haimeur A., Légaré D., Papadopoulou B., Ouellette M. (1999). Increased transport of pteridines compensates for mutations in the high affinity folate transporter and contributes to methotrexate resistance in the protozoan parasite *Leishmania tarentolae*. EMBO J..

[132] Collett C.F., Kitson C., Baker N., Steele-Stallard H.B., Santrot M.-V., Hutchinson S., Horn D., Alsford S. (2019). Chemogenomic Profiling of Antileishmanial Efficacy and Resistance in the Related Kinetoplastid Parasite Trypanosoma brucei. Antimicrob. Agents Chemother..

[133] Zhang N., Zoltner M., Leung K.-F., Scullion P., Hutchinson S., Del Pino R.C., Vincent I., Zhang Y.-K., Freund Y.R., Alley M.R.K. (2018). Host-parasite co-metabolic activation of antitrypanosomal aminomethyl-benzoxaboroles. PLoS Pathog..

[134] Macedo J., Burkard G.S., Niemann M., Barrett M.P., Vial H., Mäser P., Roditi I., Schneider A., Bütikofer P. (2015). An Atypical Mitochondrial Carrier That Mediates Drug Action in *Trypanosoma brucei*. PLoS Pathog..

[135] Hulpia F., Mabille D., Campagnaro G.D., Schumann G., Maes L., Roditi I., Hofer A., De Koning H.P., Caljon G., Van Calenbergh S. (2019). Combining tubercidin and cordycepin scaffolds results in highly active candidates to treat late-stage sleeping sickness. Nat. Commun..

[136] Dewar S., Sienkiewicz N., Ong H., Wall R.J., Horn D., Fairlamb A.H. (2016). The Role of Folate Transport in Antifolate Drug Action in Trypanosoma brucei. J. Boil. Chem..

[137] Drew M.E., Morris J.C., Wang Z., Wells L., Sanchez M., Landfear S.M., Englund P.T. (2003). The adenosine analog tubercidin inhibits glycolysis in *Trypanosoma brucei* as revealed by an RNA interference library. J. Biol. Chem..

[138] De Jesus J.B., Mesquita-Rodrigues C., Cuervo P. (2013). Proteomics Advances in the Study of *Leishmania* Parasites and Leishmaniasis. Membr. Biog..

[139] Paape D., Aebischer T. (2011). Contribution of proteomics of *Leishmania* spp. to the understanding of differentiation, drug resistance mechanisms, vaccine and drug development. J. Proteom..

[140] Sundar S., Singh B. (2018). Understanding *Leishmania* parasites through proteomics and implications for the clinic. Expert Rev. Proteom..

[141] Parthasarathy A., Kalesh K. (2020). Defeating the trypanosomatid trio: Proteomics of the protozoan parasites causing neglected tropical diseases. RSC Med. Chem..

[142] Ziegler S., Pries V., Hedberg C., Waldmann H. (2013). Target Identification for Small Bioactive Molecules: Finding the Needle in the Haystack. Angew. Chem. Int. Ed..

[143] Zhu W., Smith J.W., Huang C.-M. (2009). Mass Spectrometry-Based Label-Free Quantitative Proteomics. J. Biomed. Biotechnol..

[144] Karp N.A., Huber W., Sadowski P.G., Charles P.D., Hester S.V., Lilley K.S. (2010). Addressing accuracy and precision issues in iTRAQ quantitation. Mol. Cell. Proteom..

[145] Steen H., Pandey A. (2002). Proteomics goes quantitative: Measuring protein abundance. Trends Biotechnol..

[146] Bantscheff M., Schirle M., Sweetman G., Rick J., Kuster B. (2007). Quantitative mass spectrometry in proteomics: A critical review. Anal. Bioanal. Chem..

[147] Görg A., Drews O., Lück C., Weiland F., Weiss W. (2009). 2-DE with IPGs. Electrophoresis.

[148] Foucher A.L., Papadopoulou B., Ouellette M. (2006). Prefractionation by Digitonin Extraction Increases Representation of the Cytosolic and Intracellular Proteome of *Leishmania infantum*. J. Proteome Res..

[149] Vacchina P., Norris-Mullins B., Carlson E.S., Morales M.A. (2016). A mitochondrial HSP70 (HSPA9B) is linked to miltefosine resistance and stress response in *Leishmania donovani*. Parasites Vectors.

[150] Brotherton M.-C., Bourassa S., Légaré D., Poirier G.G., Droit A., Ouellette M. (2014). Quantitative proteomic analysis of amphotericin B resistance in *Leishmania infantum*. Int. J. Parasitol. Drugs Drug Resist..

[151] Kumar A., Sisodia B., Misra P., Sundar S., Shasany A.K., Dube A. (2010). Proteome mapping of overexpressed membrane-enriched and cytosolic proteins in sodium antimony gluconate (SAG) resistant clinical isolate of *Leishmania donovani*. Br. J. Clin. Pharmacol..

[152] Guyett P., Behera R., Ogata Y., Pollastri M., Mensa-Wilmot K. (2016). Novel Effects of Lapatinib Revealed in the African Trypanosome by Using Hypothesis-Generating Proteomics and Chemical Biology Strategies. Antimicrob. Agents Chemother..

[153] Moreira D.D.S., Pescher P., Laurent C., Lenormand P., Späth G.F., Murta S.M.F. (2015). Phosphoproteomic analysis of wild-type and antimony-resistant *Leishmania braziliensis* lines by 2D-DIGE technology. Proteomics.

[154] Garg G., Ali V., Singh K., Gupta P., Ganguly A., Sahasrabuddhe A.A., Das P. (2019). Quantitative secretome analysis unravels new secreted proteins in Amphotericin B resistant *Leishmania donovani*. J. Proteom..

[155] Vincent I., Racine G., Légaré D., Ouellette M. (2015). Mitochondrial Proteomics of Antimony and Miltefosine Resistant *Leishmania infantum*. Proteomes.

[156] Tasbihi M., Shekari F., Hajjaran H., Khanmohammadi M., Hadighi R. (2020). Comparative mitochondrial proteomics of *Leishmania tropica* clinical isolates resistant and sensitive to meglumine antimoniate. Parasitol. Res..

[157] Atwood J.A., Weatherly D.B., Minning T.A., Bundy B., Cavola C., Opperdoes F., Orlando R., Tarleton R.L. (2005). The Trypanosoma cruzi Proteome. Science.

[158] Biyani N., Madhubala R. (2012). Quantitative proteomic profiling of the promastigotes and the intracellular amastigotes of *Leishmania donovani* isolates identifies novel proteins having a role in *Leishmania* differentiation and intracellular survival. Biochim. Biophys. Acta Proteins Proteom..

[159] Magalhaes A., Queiroz R., Bastos I.M.D., Santana J., De Sousa M.V., Ricart C., Charneau S. (2015). Comparative Two-Dimensional Gel Electrophoresis of *Trypanosoma cruzi* Mammalian-Stage Forms in an Alkaline pH Range. Protein Pept. Lett..

[160] Veras P.S.T., De Menezes J.P.B., De Menezes J.B. (2016). Using Proteomics to Understand How *Leishmania* Parasites Survive inside the Host and Establish Infection. Int. J. Mol. Sci..

[161] Scheltema R.A., Decuypere S., T’Kindt R., Dujardin J.-C., Coombs G.H., Breitling R. (2010). The potential of metabolomics for *Leishmania* research in the post-genomics era. Parasitology.

[162] Drummelsmith J., Girard I., Trudel N., Ouellette M. (2004). Differential Protein Expression Analysis of *Leishmania major* Reveals Novel Roles for Methionine Adenosyltransferase and S-Adenosylmethionine in Methotrexate Resistance. J. Boil. Chem..

[163] Matrangolo F.S., Liarte D.B., Andrade L.C., De Melo M.F., Andrade J.M., Ferreira R.F., Santiago A.S., Pirovani C.P., Silva-Pereira R.A., Murta S.M.F. (2013). Comparative proteomic analysis of antimony-resistant and -susceptible *Leishmania braziliensis* and *Leishmania infantum* chagasi lines. Mol. Biochem. Parasitol..

[164] Carnielli J.B.T., De Andrade H.M., Pires S.F., Chapeaurouge A.D., Perales J., Monti-Rocha R., Carvalho S.F., Ribeiro L.P., Dietze R., Figueiredo S.G. (2014). Proteomic analysis of the soluble proteomes of miltefosine-sensitive and -resistant *Leishmania infantum* chagasi isolates obtained from Brazilian patients with different treatment outcomes. J. Proteom..

[165] El Fadili K., Drummelsmith J., Roy G., Jardim A., Ouellette M. (2009). Down regulation of KMP-11 in *Leishmania infantum* axenic antimony resistant amastigotes as revealed by a proteomic screen. Exp. Parasitol..

[166] Sharma S., Singh G., Chavan H., Dey C.S., Chadha G.S. (2009). Proteomic analysis of wild type and arsenite-resistant *Leishmania donovani*. Exp. Parasitol..

[167] Singh A., Roberts S., Ullman B., Madhubala R. (2014). A quantitative proteomic screen to identify potential drug resistance mechanism in α-difluoromethylornithine (DFMO) resistant *Leishmania donovani*. J. Proteom..

[168] Veronica J., Chandrasekaran S., Dayakar A., Devender M., Prajapati V.K., Sundar S., Maurya R. (2019). Iron superoxide dismutase contributes to miltefosine resistance in *Leishmania donovani*. FEBS J..

[169] Singh G., Chavan H., Dey C.S., Chadha G.S. (2008). Proteomic analysis of miltefosine-resistant Leishmania reveals the possible involvement of eukaryotic initiation factor 4A (eIF4A). Int. J. Antimicrob. Agents.

[170] Chawla B., Jhingran A., Panigrahi A., Stuart K.D., Madhubala R. (2011). Paromomycin Affects Translation and Vesicle-Mediated Trafficking as Revealed by Proteomics of Paromomycin–Susceptible–Resistant Leishmania donovani. PLoS ONE.

[171] Vergnes B., Gourbal B., Girard I., Sundar S., Drummelsmith J., Ouellette M. (2006). A Proteomics Screen Implicates HSP83 and a Small Kinetoplastid Calpain-related Protein in Drug Resistance in *Leishmania donovani* Clinical Field Isolates by Modulating Drug-induced Programmed Cell Death. Mol. Cell. Proteom..

[172] Biyani N., Singh A., Mandal S., Chawla B., Madhubala R. (2011). Differential expression of proteins in antimony-susceptible and -resistant isolates of *Leishmania donovani*. Mol. Biochem. Parasitol..

[173] Imbert L., Cojean S., Libong D., Chaminade P., Loiseau P.M. (2014). Sitamaquine-resistance in *Leishmania donovani* affects drug accumulation and lipid metabolism. Biomed. Pharmacother..

[174] Zarean M., Maraghi S., Hajjaran H., Mohebali M., Feiz-Hadad M.H., Assarehzadegan M.A. (2015). Comparison of Proteome Profiling of Two Sensitive and Resistant Field Iranian Isolates of *Leishmania major* to Glucantime^®^ by 2-Dimensional Electrophoresis. Iran. J. Parasitol..

[175] Walker J., Gongora R., Vasquez J.-J., Drummelsmith J., Burchmore R., Roy G., Ouellette M., Gomez M.A., Saravia N.G. (2012). Discovery of factors linked to antimony resistance in *Leishmania panamensis* through differential proteome analysis. Mol. Biochem. Parasitol..

[176] Hajjaran H., Azarian B., Mohebali M., Hadighi R., Assareh A., Vaziri B. (2012). Comparative proteomics study on meglumine antimoniate sensitive and resistant *Leishmania tropica* isolated from Iranian anthroponotic cutaneous leishmaniasis patients. East. Mediterr. Heal. J..

[177] Foucher A.L., McIntosh A., Douce G., Wastling J., Tait A., Turner C. (2006). A proteomic analysis of arsenical drug resistance in *Trypanosoma brucei*. Proteomics.

[178] Sullenberger C., Pique D., Ogata Y., Mensa-Wilmot K. (2017). AEE788 Inhibits Basal Body Assembly and Blocks DNA Replication in the African Trypanosome. Mol. Pharmacol..

[179] Zoltner M., Campagnaro G.D., Taleva G., Burrell A., Cerone M., Leung K.-F., Achcar F., Horn D., Vaughan S., Gadelha C. (2020). Suramin exposure alters cellular metabolism and mitochondrial energy production in African trypanosomes. J. Boil. Chem..

[180] Andrade H., Murta S.M.F., Chapeaurouge A., Perales J., Nirdé P., Romanha Á.J. (2008). Proteomic Analysis of *Trypanosoma cruzi* Resistance to Benznidazole. J. Proteome Res..

[181] Brunoro G.V.F., Faça V.M., Caminha M.A., Ferreira A.T.D.S., Trugilho M., De Moura K.C.G., Perales J., Valente R.H., Menna-Barreto R.F.S. (2016). Differential Gel Electrophoresis (DIGE) Evaluation of Naphthoimidazoles Mode of Action: A Study in *Trypanosoma cruzi* Bloodstream Trypomastigotes. PLoS Negl. Trop. Dis..

[182] Menna-Barreto R.F., Beghini D.G., Ferreira A.T., Pinto A.V., De Castro S.L., Perales J. (2010). A proteomic analysis of the mechanism of action of naphthoimidazoles in *Trypanosoma cruzi* epimastigotes in vitro. J. Proteom..

[183] Vieira G.A.L., Da Silva M.T.A., Regasini L.O., Cotinguiba F., Laure H.J., Rosa J.C., Furlan M., Cicarelli R.M.B. (2018). *Trypanosoma cruzi*: Analysis of two different strains after piplartine treatment. Braz. J. Infect. Dis..

[184] Lee H., Lee J.W. (2016). Target identification for biologically active small molecules using chemical biology approaches. Arch. Pharmacal Res..

[185] Kubota K., Funabashi M., Ogura Y. (2018). Target deconvolution from phenotype-based drug discovery by using chemical proteomics approaches. Biochim. Biophys. Acta Proteins Proteom..

[186] Drewes G., Knapp S. (2018). Chemoproteomics and Chemical Probes for Target Discovery. Trends Biotechnol..

[187] Sato S.-I., Murata A., Shirakawa T., Uesugi M. (2010). Biochemical Target Isolation for Novices: Affinity-Based Strategies. Chem. Boil..

[188] Kuettel S., Mosimann M., Mäser P., Kaiser M., Brun R., Scapozza L., Perozzo R. (2009). Adenosine Kinase of T. b. rhodesiense Identified as the Putative Target of 4-[5-(4-phenoxyphenyl)-2H-pyrazol-3-yl]morpholine Using Chemical Proteomics. PLoS Negl. Trop. Dis..

[189] Knockaert M., Gray N., Damiens E., Chang Y.-T., Grellier P., Grant K., Fergusson D., Mottram J.C., Soete M., Dubremetz J.F. (2000). Intracellular targets of cyclin-dependent kinase inhibitors: Identification by affinity chromatography using immobilised inhibitors. Chem. Boil..

[190] Mercer L., Bowling T., Perales J., Freeman J., Nguyen T., Bacchi C., Yarlett N., Don R., Jacobs R., Nare B. (2011). 2,4-Diaminopyrimidines as Potent Inhibitors of *Trypanosoma brucei* and Identification of Molecular Targets by a Chemical Proteomics Approach. PLoS Negl. Trop. Dis..

[191] Konziase B. (2015). Biotinylated probes of artemisinin with labeling affinity toward *Trypanosoma brucei* brucei target proteins. Anal. Biochem..

[192] Yang P.-Y., Wang M., He C.Y., Yao S.Q. (2012). Proteomic profiling and potential cellular target identification of K11777, a clinical cysteine protease inhibitor, in *Trypanosoma brucei*. Chem. Commun..

[193] Ong S.-E., Schenone M., Margolin A.A., Li X., Do K., Doud M.K., Mani D.R., Kuai L., Wang X., Wood J.L. (2009). Identifying the proteins to which small-molecule probes and drugs bind in cells. Proc. Natl. Acad. Sci. USA.

[194] Park S.B., Koh M., Park S.B. (2013). From noncovalent to covalent bonds: A paradigm shift in target protein identification. Mol. BioSyst..

[195] Yang P.-Y., Wang M., Li L., Wu H., He C.Y., Yao S.Q. (2012). Design, Synthesis and Biological Evaluation of Potent Azadipeptide Nitrile Inhibitors and Activity-Based Probes as Promising Anti-*Trypanosoma brucei* Agents. Chem. A Eur. J..

[196] Smith E., Collins I. (2015). Photoaffinity labeling in target- and binding-site identification. Futur. Med. Chem..

[197] Tulloch L., Menzies S., Fraser A.L., Gould E.R., King E.F., Zacharova M.K., Florence G.J., Smith T.K. (2017). Photo-affinity labelling and biochemical analyses identify the target of trypanocidal simplified natural product analogues. PLoS Negl. Trop. Dis..

[198] Lomenick B., Hao R., Jonai N., Chin R.M., Aghajan M., Warburton S., Wang J., Wu R.P., Gomez F., Loo J.A. (2009). Target identification using drug affinity responsive target stability (DARTS). Proc. Natl. Acad. Sci. USA.

[199] Lomenick B., Olsen R.W., Huang J. (2010). Identification of Direct Protein Targets of Small Molecules. ACS Chem. Boil..

[200] Park H., Ha J., Park S.B. (2019). Label-free target identification in drug discovery via phenotypic screening. Curr. Opin. Chem. Boil..

[201] Mateus A., Määttä T.A., Savitski M.M. (2016). Thermal proteome profiling: Unbiased assessment of protein state through heat-induced stability changes. Proteome Sci..

[202] Chang J., Kim Y., Kwon H.J. (2016). Advances in identification and validation of protein targets of natural products without chemical modification. Nat. Prod. Rep..

[203] Konziase B. (2015). Analytical purification of a 60-kDa target protein of artemisinin detected in *Trypanosoma brucei* brucei. Data Brief.

[204] Denise H., Giroud C., Barrett M.P., Baltz T. (1999). Affinity chromatography using trypanocidal arsenical drugs identifies a specific interaction between glycerol-3-phosphate dehydrogenase from *Trypanosoma brucei* and Cymelarsan. JBIC J. Boil. Inorg. Chem..

[205] Pieretti S., Haanstra J.R., Mazet M., Perozzo R., Bergamini C., Prati F., Fato R., Lenaz G., Capranico G., Brun R. (2013). Naphthoquinone Derivatives Exert Their Antitrypanosomal Activity via a Multi-Target Mechanism. PLoS Negl. Trop. Dis..

[206] Trochine A., Álvarez G., Corre S., Faral-Tello P., Duran R., Batthyány C., Cerecetto H., Gonzalez M., Robello C. (2014). Trypanosoma cruzi chemical proteomics using immobilized benznidazole. Exp. Parasitol..

[207] Wishart D.S. (2008). Applications of Metabolomics in Drug Discovery and Development. Drugs R&D.

[208] Creek D.J., Anderson J., McConville M.J., Barrett M.P. (2012). Metabolomic analysis of trypanosomatid protozoa. Mol. Biochem. Parasitol..

[209] Berg M., Vanaerschot M., Jankevics A., Cuypers B., Breitling R., Dujardin J.-C. (2013). LC-MS metabolomics from study design to data-analysis—Using a versatile pathogen as a test case. Comput. Struct. Biotechnol. J..

[210] Courant F., Antignac J.-P., Dervilly-Pinel G., Le Bizec B. (2014). Basics of mass spectrometry based metabolomics. Proteomics.

[211] Rojo D., Canuto G.A., Castilho-Martins E.A., Tavares M.F.M., Barbas C., López-Gonzálvez Á., Rivas L. (2015). A Multiplatform Metabolomic Approach to the Basis of Antimonial Action and Resistance in *Leishmania infantum*. PLoS ONE.

[212] Armitage E.G., Alqaisi A.Q.I., Godzien J., Peña I., Mbekeani A.J., Alonso-Herranz V., López-Gonzálvez Á., Martín J., Gabarró R., Denny P.W. (2018). Complex Interplay between Sphingolipid and Sterol Metabolism Revealed by Perturbations to the *Leishmania* Metabolome Caused by Miltefosine. Antimicrob. Agents Chemother..

[213] Canuto G.A., Castilho-Martins E.A., Tavares M.F.M., Rivas L., Barbas C., López-Gonzálvez Á. (2014). Multi-analytical platform metabolomic approach to study miltefosine mechanism of action and resistance in *Leishmania*. Anal. Bioanal. Chem..

[214] T’Kindt R., Jankevics A., Scheltema R.A., Zheng L., Watson D.G., Dujardin J.-C., Breitling R., Coombs G.H., Decuypere S. (2010). Towards an unbiased metabolic profiling of protozoan parasites: Optimisation of a Leishmania sampling protocol for HILIC-orbitrap analysis. Anal. Bioanal. Chem..

[215] Creek D.J. (2013). Determination of antiprotozoal drug mechanisms by metabolomics approaches. Parasitology.

[216] Canuto G.A., Castilho-Martins E.A., Tavares M., López-Gonzálvez Á., Rivas L., Barbas C. (2012). CE-ESI-MS metabolic fingerprinting of *Leishmania* resistance to antimony treatment. Electrophoresis.

[217] Vincent I., Weidt S., Rivas L., Burgess K., Smith T.K., Ouellette M. (2014). Untargeted metabolomic analysis of miltefosine action in *Leishmania infantum* reveals changes to the internal lipid metabolism. Int. J. Parasitol. Drugs Drug Resist..

[218] Dumetz F., Imamura H., Sanders M., Seblova V., Myskova J., Pescher P., Vanaerschot M., Meehan C., Cuypers B., De Muylder G. (2017). Modulation of Aneuploidy in *Leishmania donovani* during Adaptation to Different In Vitro and In Vivo Environments and Its Impact on Gene Expression. mBio.

[219] Berg M., García-Hernández R., Cuypers B., Vanaerschot M., Manzano J.I., Poveda J.A., Ferragut J.A., Castanys S., Dujardin J.-C., Gamarro F. (2015). Experimental Resistance to Drug Combinations in *Leishmania donovani*: Metabolic and Phenotypic Adaptations. Antimicrob. Agents Chemother..

[220] Vincent I., Creek D.J., Watson D.G., Kamleh M.A., Woods D.J., Wong P.E., Burchmore R., Barrett M.P. (2010). A Molecular Mechanism for Eflornithine Resistance in African Trypanosomes. PLoS Pathog..

[221] Ali J.A.M., Creek D.J., Burgess K., Allison H.C., Field M.C., Mäser P., De Koning H.P. (2012). Pyrimidine salvage in *Trypanosoma brucei* bloodstream forms and the trypanocidal action of halogenated pyrimidines. Mol. Pharmacol..

[222] Armitage E.G., Godzien J., Peña I., López-Gonzálvez Á., Angulo S., Gradillas A., Alonso-Herranz V., Martín J., Fiandor J.M., Barrett M.P. (2018). Metabolic Clustering Analysis as a Strategy for Compound Selection in the Drug Discovery Pipeline for Leishmaniasis. ACS Chem. Boil..

[223] Steketee P., Vincent I., Achcar F., Giordani F., Kim D.-H., Creek D.J., Freund Y., Jacobs R., Rattigan K., Horn D. (2018). Benzoxaborole treatment perturbs S-adenosyl-L-methionine metabolism in *Trypanosoma brucei*. PLoS Negl. Trop. Dis..

[224] Hennig K., Abi-Ghanem J., Bunescu A., Meniche X., Biliaut E., Ouattara A.D., Lewis M.D., Kelly J.M., Braillard S., Courtemanche G. (2019). Metabolomics, lipidomics and proteomics profiling of myoblasts infected with Trypanosoma cruzi after treatment with different drugs against Chagas disease. Metabolomics.

[225] Almugadam S.H., Trentini A., Maritati M., Contini C., Rugna G., Bellini T., Manfrinato M.C., Dallocchio F., Hanau S. (2018). Influence of 6-aminonicotinamide (6AN) on *Leishmania* promastigotes evaluated by metabolomics: Beyond the pentose phosphate pathway. Chem. Interact..

[226] Lima M.L., Abengózar M.A., Nácher-Vázquez M., Martínez-Alcázar M.P., Barbas C., Tempone A.G., López-Gonzálvez Á., Rivas L. (2018). Molecular Basis of the Leishmanicidal Activity of the Antidepressant Sertraline as a Drug Repurposing Candidate. Antimicrob. Agents Chemother..

[227] Alzahrani K.J., Ali J.A., Eze A.A., Looi W.L., Tagoe D.N., Creek D.J., Barrett M.P., De Koning H.P. (2017). Functional and genetic evidence that nucleoside transport is highly conserved in *Leishmania* species: Implications for pyrimidine-based chemotherapy. Int. J. Parasitol. Drugs Drug Resist..

[228] Mbekeani A., Jones R., Llorens M.B., Elliot J., Regnault C., Barrett M., Steele J., Kebede B., Wrigley S., Evans L. (2019). Mining for natural product antileishmanials in a fungal extract library. Int. J. Parasitol. Drugs Drug Resist..

[229] Rebello K.M., Andrade-Neto V.V., Zuma A.A., Motta M.C.M., Gomes C.R.B., De Souza M.V.N., Atella G.C., Branquinha M.H., Santos A.L.S., Torres-Santos E.C. (2018). Lopinavir, an HIV-1 peptidase inhibitor, induces alteration on the lipid metabolism of *Leishmania amazonensis* promastigotes. Parasitology.

[230] Vincent I., Creek D.J., Burgess K., Woods D.J., Burchmore R., Barrett M.P. (2012). Untargeted Metabolomics Reveals a Lack Of Synergy between Nifurtimox and Eflornithine against *Trypanosoma brucei*. PLoS Negl. Trop. Dis..

[231] Trochine A., Creek D.J., Faral-Tello P., Barrett M.P., Robello C. (2014). Benznidazole Biotransformation and Multiple Targets in *Trypanosoma cruzi* Revealed by Metabolomics. PLoS Negl. Trop. Dis..

[232] Schirle M., Jenkins J. (2016). Identifying compound efficacy targets in phenotypic drug discovery. Drug Discov. Today.

[233] Wyatt P.G., Gilbert I.H., Read K.D., Fairlamb A.H. (2011). Target Validation: Linking Target and Chemical Properties to Desired Product Profile. Curr. Top. Med. Chem..

[234] Brunoro G.V.F., Caminha M.A., Ferreira A.T.D.S., Vos R.A., Carvalho P.C., Perales J., Valente R.H., Menna-Barreto R.F.S., Le Prevost F.D.V. (2015). Reevaluating the *Trypanosoma cruzi* proteomic map: The shotgun description of bloodstream trypomastigotes. J. Proteom..

